# Mental Health Among Employed Family Caregivers: A Gender-Based Analysis on the Role of Workplace Support

**DOI:** 10.1093/geroni/igaa057.242

**Published:** 2020-12-16

**Authors:** Lun Li, Yeonjung Lee, Daniel W L Lai

**Affiliations:** 1 Simon Fraser University, Vancouver, British Columbia, Canada; 2 University of Calgary, Calgary, Alberta, Canada; 3 The Hong Kong Polytechnic University, Kowloon, China

## Abstract

Compared to men, women undertake more family caregiving responsibilities, and thus take more toll in health and wellbeing when they are employed. The current study examined the gender difference in mental health among employed family caregivers, focusing on the role of workplace support in balancing work and caregiving roles. Guided by the social role theory and the moderated-mediation model of employment adjustment and mental health, we analyzed a nationally representative data from the 2012 Canada General Social Survey - Caregiving and Care Receiving with a sample of 2,426 participants selected. Moderated-mediation analysis based on the SPSS macro PROCESS 3.3 was used. Women employed family caregivers are more likely to undertake higher intensive caregiving, make more employment adjustment, and further report worse mental health status than their men counterparts. Gender difference was apparent in regards to the workplace support. For women, the moderating effect of workplace support is significant only when there are at least 5 different types of workplace support available at their workplaces, while for men, the moderating effect is significant when there are at least 2-3 different types of workplace support available. Women employed family caregivers experience worse mental health when employment adjustment is needed for their care responsibility. Findings have implications for providing workplace support for family caregivers given that women benefit less from workplace support compared to men. Further study is needed to explore the impact of various types of workplace support for women employed family caregivers, and to provide tailored support to them.

